# Reduced spinal microglial activation and neuropathic pain after nerve injury in mice lacking all three nitric oxide synthases

**DOI:** 10.1186/1744-8069-7-50

**Published:** 2011-07-14

**Authors:** Kazuya Kuboyama, Makoto Tsuda, Masato Tsutsui, Yumiko Toyohara, Hidetoshi Tozaki-Saitoh, Hiroaki Shimokawa, Nobuyuki Yanagihara, Kazuhide Inoue

**Affiliations:** 1 Department of Molecular and System Pharmacology, Graduate School of Pharmaceutical Sciences, University of Kyushu, Fukuoka, Japan; 2 Department of Pharmacology, Graduate School of Medicine, University of the Ryukyu, Okinawa, Japan; 3 Department of Pharmacology, University of Occupational & Environmental Health, School of Medicine, Fukuoka, Japan; 4 Department of Cardiovascular Medicine, Graduate School of Medicine, Tohoku University, Miyagi, Japan

## Abstract

**Background:**

Several studies have investigated the involvement of nitric oxide (NO) in acute and chronic pain using mice lacking a single NO synthase (NOS) gene among the three isoforms: neuronal (nNOS), inducible (iNOS) and endothelial (eNOS). However, the precise role of NOS/NO in pain states remains to be determined owing to the substantial compensatory interactions among the NOS isoforms. Therefore, in this study, we used mice lacking all three NOS genes (*n/i/eNOS^-/-^*mice) and investigated the behavioral phenotypes in a series of acute and chronic pain assays.

**Results:**

In a model of tissue injury-induced pain, evoked by intraplantar injection of formalin, both *iNOS^-/-^*and *n/i/eNOS^-/-^*mice exhibited attenuations of pain behaviors in the second phase compared with that in wild-type mice. In a model of neuropathic pain, nerve injury-induced behavioral and cellular responses (tactile allodynia, spinal microglial activation and Src-family kinase phosphorylation) were reduced in *n/i/eNOS^-/-^*but not *iNOS^-/-^*mice. Tactile allodynia after nerve injury was improved by acute pharmacological inhibition of all NOSs and nNOS. Furthermore, in MG-5 cells (a microglial cell-line), interferon-γ enhanced NOSs and Mac-1 mRNA expression, and the Mac-1 mRNA increase was suppressed by L-NAME co-treatment. Conversely, the NO donor, sodium nitroprusside, markedly increased mRNA expression of Mac-1, interleukin-6, toll-like receptor 4 and P2X4 receptor.

**Conclusions:**

Our results provide evidence that the NOS/NO pathway contributes to behavioral pain responses evoked by tissue injury and nerve injury. In particular, nNOS may be important for spinal microglial activation and tactile allodynia after nerve injury.

## Background

Acute pain can act as an early warning device that alerts us to the presence of damaging stimuli, e.g. trauma, chemical irritation and thermal stimuli. This pain usually goes away soon after the noxious stimulus is removed. In contrast, chronic pain persists for a long time, and is categorized into inflammatory pain and neuropathic pain. Neuropathic pain, resulting from peripheral nerve injury, is characterized by spontaneous pain, hyperalgesia and allodynia [1]. Allodynia is nearly always resistant to known treatments such as non-steroidal anti-inflammatory drugs (NSAIDs) or even opioids. After nerve injury, spinal microglia that are the main immunocompetent cells in the central nervous system transform into an activated form. Reactive microglia change their morphology and number, and express several molecules such as interleukin (IL)-1β, IL-6, interferon-γ (IFN-γ), toll-like receptor 4 (TLR4), P2X4 receptor and nitric oxide synthases (NOSs) [2-6]. Therefore, glial cells, especially microglia, have received much attention as a new therapeutic target for the treatment of neuropathic pain. However, the mechanism of neuropathic pain is not fully understood, and there are currently no effective remedies.

Nitric oxide (NO) is a free radical that produces a variety of biological actions under physiological and pathological conditions [7-9] and is synthesized by three isoforms of NOS: neuronal NOS (nNOS), inducible NOS (iNOS) and endothelial NOS (eNOS) [10-12]. Previous studies have shown the involvement of NOSs and NO in acute and chronic pain using pharmacological procedures or mice lacking a single NOS gene among the three isoforms. For instance, intraperitoneal administration of a NO precursor or a NO donor potentiates thermal hyperalgesia and tactile allodynia in neuropathic rats [13]. A NOS inhibitor effectively improves tactile allodynia by tight ligation of the fifth and sixth lumbar spinal nerves in rats [14]. Moreover, *nNOS^-/-^*mice fail to display nerve injury-induced tactile hypersensitivity [15]. In contrast, *iNOS^-/-^*mice do not affect nerve injury-induced thermal allodynia [16]. However, because there is a mechanism to compensate for the quantity of NO in NOSs [17-19], the precise role of endogenous NO still remains to be determined.

In this study, we sought to investigate the role of the NOS/NO pathway in models of acute and chronic pain using mice lacking the iNOS gene (*iNOS^-/-^*mice) and mice lacking all three NOS genes (nNOS, iNOS and eNOS *triply-NOS^-/-^*mice: *n/i/eNOS^-/-^*mice) [20]. Here, we demonstrate that endogenous NO is a critical player for inducing microglial activation in the spinal cord and in pain hypersensitivity after peripheral nerve injury. Furthermore, by using an MG-5 microglial cell-line, we show that NO induces microglial activation. Taken together, inhibition of NOS/NO signaling may be a viable therapeutic strategy for treating neuropathic pain.

## Results

### Effect of NOS gene knockout on tissue injury- and chemical-induced pain and acute mechanical and thermal pain behavior responses

**Figure 1:**
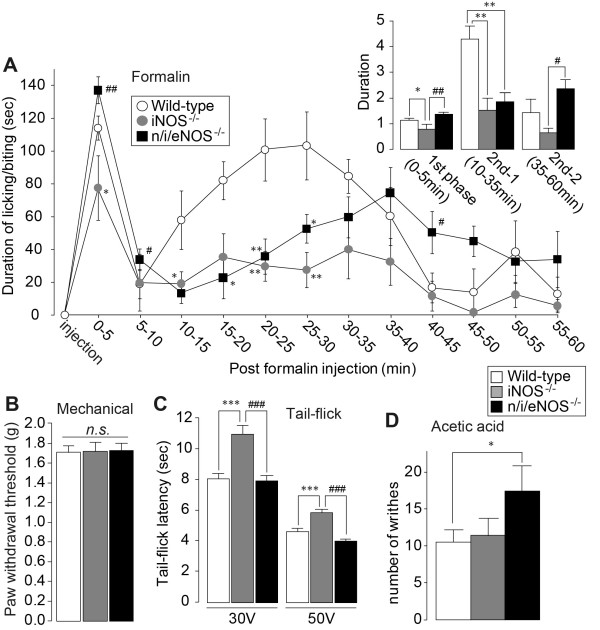
**Acute mechanical and thermal pain and inflammatory- and chemical-induced pain in *iNOS^-/-^*and *n/i/eNOS^-/-^*mice**. (A) Formalin test. Mice were injected intraplantarly with formalin (5% (v/v), 20 μL). Values represent the duration (sec) of licking and biting responses for each 5-min interval, from 0-5 min (1st phase), for 10-35 min (2nd-1 phase) and for 35-60 min (2nd-2 phase) (*n *= 8 animals/group). (B) Innocuous mechanical stimuli test. Values indicate the threshold (g) to elicit paw withdrawal behavior in response to mechanical stimuli (WT mice and *n/i/eNOS*^**-/-**^mice, *n *= 9, *iNOS*^**-/-**^mice *n *= 8 animals) (C) Tail-flick test. Values represent the latency (sec) for animals to flick their tail away from the heat source (*n *= 8 animals/group) (D) Visceral pain in response to acetic acid (0.8% (v/v)). Values represent the numbers of abdominal stretches (writhes) (*n *= 8 animals/group). All data are presented as means ± SEM. Statistical analysis were determined by two-way repeated ANOVA (A) or two-way ANOVA (B, C, D) with Tukey's test, *P < 0.05, **P < 0.01 vs. WT mice, #*P *< 0.05, ##*P *< 0.01 vs. *iNOS*^**-/-**^mice.

First, to examine the role of NOSs in physiological pain, we assessed the acute and chronic pain responses following injection of formalin into the hindpaw and intraperitoneal injection with acetic acid. After formalin injection, wild-type (WT) mice displayed biphasic licking and biting behaviors: the first acute pain phase started immediately after injection and lasted for 5 min, and the second persistent pain phase lasted for 60 min (see Figure 1A). In contrast to WT mice, the magnitude of biphasic behaviors were significantly suppressed in *iNOS^-/-^*mice (first phase, *P *< 0.05; second phase, *P *< 0.05-0.01 vs. WT mice; Figure 1A). Meanwhile, *n/i/eNOS^-/-^*mice resulted in a reduction of pain-related behavior in only the second phase as compared with that in WT mice (second phase, *P *< 0.05-0.01 vs. WT mice; Figure 1A). Interestingly, when compared with *iNOS^-/-^*mice but not with WT mice, *n/i/eNOS^-/-^*mice showed enhanced nociceptive responses in the first phase and late second phase (after 35-60 min of formalin injection) (first phase, *P *< 0.01; late second phase, *P *< 0.05 vs. *iNOS^-/-^*mice; first phase, *P *= 0.060; late second phase, *P *= 0.143 vs. WT mice; Figure 1A). In a test of innocuous mechanical stimuli applied by von Frey filaments, both *iNOS^-/-^*and *n/i/eNOS^-/-^*mice were indistinguishable from WT mice in terms of their paw withdrawal thresholds (PWTs) (Figure 1B). In tail-flick tests, the latencies for mice to flick their tails away from radiant heat at both 30 V and 50 V were not different between WT mice and *n/i/eNOS^-/-^*mice, but only *iNOS^-/-^*mice showed a significant endurance of the noxious thermal stimuli as compared with others (*P *< 0.001 vs. WT mice and *n/i/eNOS^-/-^*mice; Figure 1C). In *n/i/eNOS^-/-^*but not in *iNOS^-/-^*mice, the frequency of abdominal writhing behaviors in response to intraperitoneal injection with acetic acid, a model of chemical-induced visceral pain, increased when compared with WT mice (*P *< 0.05 vs. WT mice; Figure 1D).

### Effect of NOS gene knockout on nerve injury-induced tactile allodynia and spinal microglial activation

**Figure 2:**
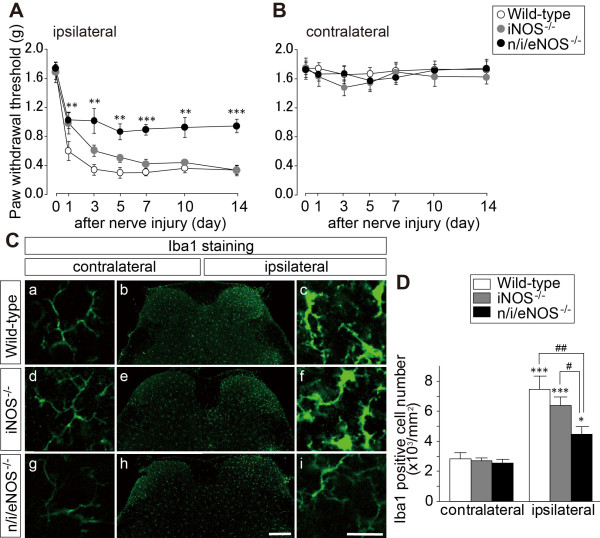
**Spinal nerve injury-induced tactile allodynia and microglial activation in *iNOS^-/-^*and *n/i/eNOS^-/-^*mice**. PWTs (A; ipsilateral hindpaw, B; contralateral hindpaw) of WT mice (*n *= 9), *iNOS***^-/- ^**mice (*n *= 8) and *n/i/eNOS*^**-/-**^mice (*n *= 9) before (0) and 1, 3, 7, 10 and 14 days after fifth lumbar spinal nerve injury. All data are presented as means ± SEM. Statistical analysis were determined by two-way repeated ANOVA with Tukey's test, *P < 0.05, **P < 0.01, *** *P *< 0.001 vs. WT mice. (C) Photomicrographs show immunostaining with Iba1, a marker of microglia, in the fifth lumbar spinal dorsal horn of WT mice (a-c), *iNOS*^**-/-**^mice (d-f) and *n/i/eNOS*^**-/-**^mice (g-i) 14 days after nerve injury. Scale bars: 200 μm (b, e, h) and 20 μm (a, c, d, f, g, i) (D) The number of Iba1**^+^**microglia in the spinal dorsal horn (*n *= 4 animals/group). All data are presented as means ± SEM. Statistical analysis were determined by two-way ANOVA with Tukey's test, *P < 0.05, ***P < 0.001 vs. contralateral, #*P *< 0.05, ##*P *< 0.01.

In WT mice, withdrawal threshold of the hindpaw ipsilateral to the nerve injury markedly decreased (Figure 2A). While *iNOS^-/-^*mice displayed a similar pain response to WT mice after nerve injury, the nerve injury-induced decrease in PWT was significantly suppressed in *n/i/eNOS^-/-^*mice when compared with WT mice (day 1, 3, 5 and 10, *P *< 0.01; day 7 and 14, *P *< 0.001 vs. WT mice; Figure 2A). In the contralateral hindpaw, both *iNOS^-/-^*and *n/i/eNOS^-/-^*mice had a similar PWT to that in WT mice (Figure 2B).

Spinal microglia are known to become activated after nerve injury and play an important role in subsequent pain hypersensitivity [2,21,22]. To examine the role of NOSs in microglial activation, we performed immunostaining for ionized calcium binding adaptor molecule 1 (Iba1), a marker of microglia, in the spinal dorsal horn. On day 14 after nerve injury, Iba1 immunoreactivity was markedly enhanced in the ipsilateral dorsal horn in WT mice as compared with that in the contralateral dorsal horn. In the ipsilateral dorsal horn, the changes in morphology of microglial cells after nerve injury (hypertrophy of cell body and retraction of processes) were observed in WT mice and in *iNOS^-/-^*mice but were much less in *n/i/eNOS^-/-^*mice (Figure 2C). The numbers of ipsilateral Iba1^+ ^microglial cells after nerve injury increased in all three types of mice, but the increase in *n/i/eNOS^-/-^*mice was significantly less when compared with those in WT mice (*P *< 0.01) and in *iNOS^-/-^*mice (*P *< 0.05; Figure 2D). Neither morphology nor number of Iba1^+ ^cells were altered in any three lines of mice in the contralateral dorsal horn (Figure 2C, D).

### Suppression in activation of Src-family kinases and expression of IL-6 and TLR4 mRNA after spinal nerve injury in *n/i/eNOS^-/-^*mice

**Figure 3:**
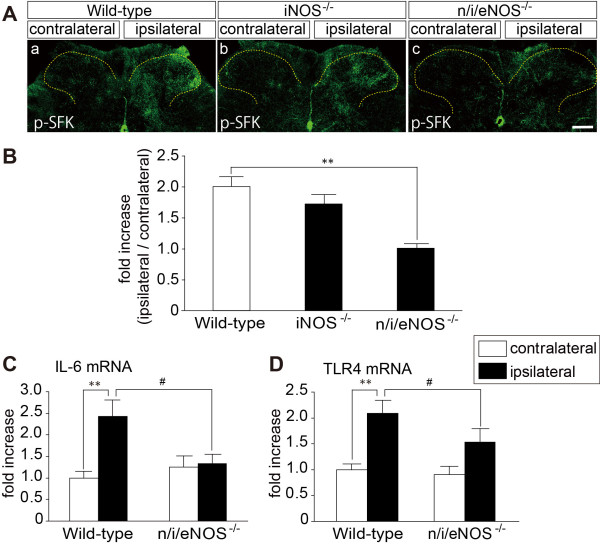
**Reduction in the amount of p-SFKs in *iNOS^-/-^*and *n/i/eNOS^-/-^*mice, and changes in IL-6 and TLR4 mRNA expression levels in *n/i/eNOS^-/-^*mice**. (A) Photomicrographs show immunostaining for phosphorylated-Src family kinases (p-SFKs) in the fifth lumbar spinal dorsal horn of WT mice (a), *iNOS^-/-^*mice (b) and *n/i/eNOS^-/-^*mice (c) 14 days after spinal nerve injury. Scale bar: 200 μm. (B) Analysis of p-SFKs immunoreactivity in the spinal dorsal horn of WT mice, *iNOS*^**-/-**^mice and *n/i/eNOS*^**-/-**^mice (*n *= 3 animals/group). (C, D) Levels of interleukin-6 (IL-6) and toll-like receptor 4 (TLR4) mRNA expression. Expression of IL-6 and TLR4 were significantly increased in the ipsilateral spinal cord when compared with the contralateral spinal cord in WT mice (*n *= 5) 14 days after nerve injury. In *n/i/eNOS*^**-/-**^mice (*n *= 4), IL-6 (C) and TLR4 (D) mRNA expression levels were significantly attenuated when compared with WT mice. All data are presented as a mean ± SEM. Statistical analysis was determined by one-way ANOVA with Tukey's test, **P < 0.01, #*P *< 0.05.

Src-family kinases (SFKs) have been reported to be activated in microglia in the spinal dorsal horn after nerve injury and implicated in neuropathic pain [23]. Increased immunoreactivity of phosphorylated-SFKs (p-SFKs), an active form, in the ipsilateral dorsal horn of WT mice was observed on day 14 after spinal nerve injury. Nerve injury-induced SFK phosphorylation was markedly suppressed in *n/i/eNOS^-/-^*but not *iNOS^-/-^*mice (Figure 3A, B). Moreover, expression of inflammatory cytokines and plasma membrane receptors, such as IL-6, IL-1β, TNF-α and TLR4, has been reported to be upregulated in the spinal cord after nerve injury [3-5]. Interestingly, the increase in IL-6 and TLR4 mRNA levels, but not IL-1β and TNF-α (data not shown), were significantly reduced in *n/i/eNOS^-/-^*mice (*P *< 0.05 both vs. WT mice; Figure 3C, D). We have now examined the levels of P2X4R mRNA expression in the spinal cord on day 14 after nerve injury by a real-time PCR analysis. In WT mice, P2X4R mRNA level was increased in 1.61 ± 0.51-fold in the ipsilateral spinal cord as compared with that in the contralateral side. Similarly, 1.61 ± 0.46-fold increase of P2X4R mRNA was observed in *iNOS^-/-^*mice. The upregulation of P2X4R mRNA expression was tendency to be suppressed in *n,i, eNOS^-/-^*mice (0.77 ± 0.42-fold increase; *P *= 0.282 vs. WT mice, *P *= 0.247 vs. *iNOS^-/-^*mice, *n *= 4).

### Pharmacological inhibition of nNOS suppresses tactile allodynia after spinal nerve injury

**Figure 4:**
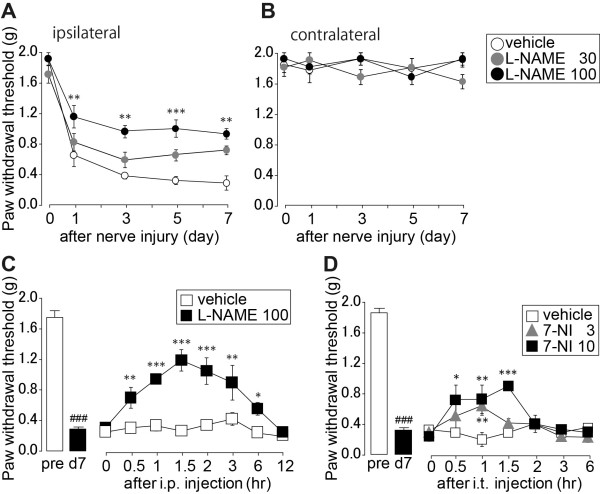
**Pharmacological inhibition of NOS/NO suppressed tactile allodynia after spinal nerve injury**. (A, B) Effect of L-NAME, a non-selective NOS inhibitor. PWTs (A; ipsilateral hindpaw, B; contralateral hindpaw) following intraperitoneal administration of L-NAME to rats once daily before (0) and 1, 3, 5 and 7 days after fifth lumbar spinal nerve injury (*n *= 6 animals/group). (C) Effect of intraperitoneal (i.p.) administration of L-NAME on the decrease in PWTs after 7 days of spinal nerve injury (*n *= 4 animals/group). (D) Effect of intraperitoneal (i.t.) administration of 7-nitroindazole (7-NI), a selective nNOS inhibitor, on the decrease in PWTs after 7 days of spinal nerve injury (*n *= 4 animals/group). All data are presented as a mean ± SEM. Statistical analysis were determined by one-way repeated ANOVA (C) or two-way repeated ANOVA (A, B, D) with Tukey's test, *P < 0.05, **P < 0.01, ***P < 0.001 vs. vehicle treatment, ###*P *< 0.001 vs. pre measurement.

We tested the effects of NOS inhibitors on the development and maintenance of nerve injury-induced tactile allodynia. Intraperitoneal administration of L-N^G^-nitro-arginine methyl ester (L-NAME), a non-specific NOS inhibitor, to rats once daily for 7 days, significantly suppressed the decrease in ipsilateral PWT after nerve injury in a dose-dependent manner without an effect on the contralateral hindpaw (day 1, 3 and 7, *P *< 0.001; day 5, *P *< 0.01 100 mg/kg L-NAME vs. vehicle group in ipsilateral; Figure 4A, B). Furthermore, acute intraperitoneal administration of L-NAME and acute intrathecal administration of 7-nitroindazole, a specific nNOS inhibitor, reversed tactile allodynia on day 7 after nerve injury (*P *< 0.05-0.001 both vs. vehicle group; Figure 4C, D).

### IFN-γ activated in microglia via NOS/NO

**Figure 5:**
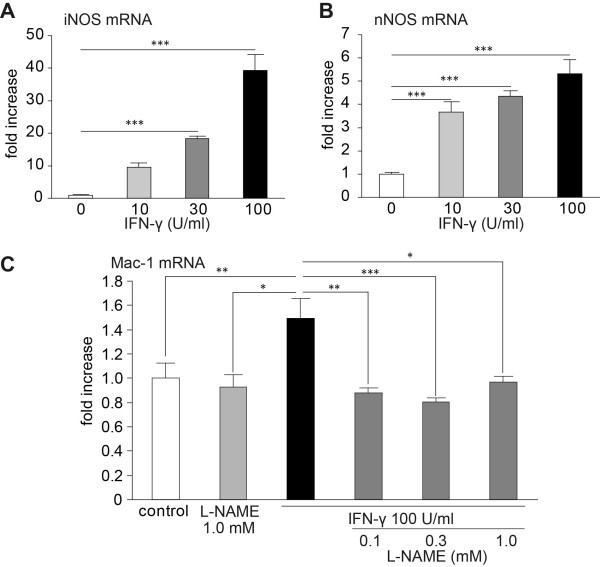
**Increased expression of iNOS, nNOS and Mac-1 mRNA in microglial cell line cultures**. Expression levels of iNOS (A) and nNOS (B) mRNA in MG-5 microglial cell line cultures. These mRNA expression levels significantly increased following treatment with interferon-γ (IFN-γ) in a dose-dependent manor (*n *= 6 samples/group). In addition, (C) the expression levels of Mac-1 mRNA were significantly increased following IFN-γ exposure. This increase was attenuated by co-treatment with L-NAME (n = 6 samples/group). All data are presented as a mean ± SEM. Statistical analysis was determined by one-way ANOVA with Tukey's test, *P < 0.05, **P < 0.01, ***P < 0.001.

**Figure 6:**
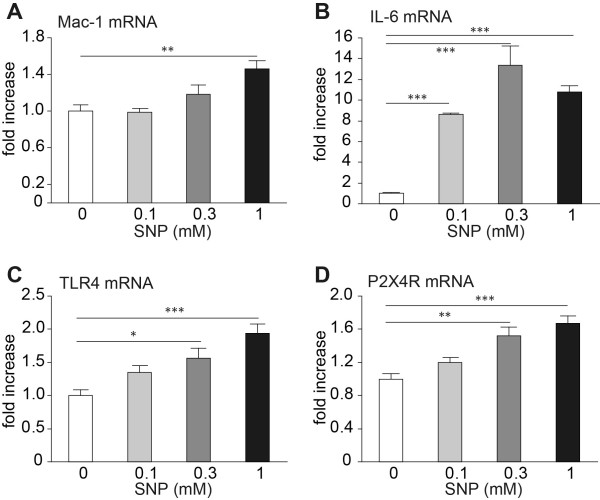
**Microglial activation, and increased IL-6, TLR4 and P2X4 receptor mRNA expression levels via NO**. Expression levels of Mac-1 (A), interleukin-6 (IL-6; B), toll-like receptor 4 (TLR4; C) and P2X4 receptor (P2X4R; D) mRNA in MG-5 microglial cell line cultures. mRNA expression levels were significant increased by treatment with sodium nitroprusside (SNP), a NO donor (*n *= 6 samples/group). All data are presented as a mean ± SEM. Statistical analysis was determined by one-way ANOVA with Tukey's test, *P < 0.05, **P < 0.01, ***P < 0.001.

IFN-γ levels are known to increase in the spinal cord after spinal nerve injury, and are implicated in microglial activation and tactile allodynia [3,24]. To investigate the involvement of NOS/NO signaling in IFN-γ-induced microglial activation, we examined the effect of IFN-γ on microglial transcriptional alterations using MG-5 cells, a mouse microglial cell-line [25]. IFN-γ induced a significant increase in the expression of iNOS and nNOS mRNA in a dose-dependent manner in MG-5 cells (39.3 ± 5.0-fold of iNOS and 5.32 ± 0.61-fold of nNOS mRNA in 100 U/mL IFN-γ as compared with non-treatment; *P *< 0.001 both; Figure 5A, B). Additionally, in IFN-γ-stimulated MG-5 cells, Mac-1 mRNA expression levels, which are known to be upregulated in activated microglia, were significantly increased by 1.50 ± 0.16- and 1.61 ± 0.17-fold as compared with non-treated MG-5 cells (*P *< 0.01; Figure 5C). This IFN-γ-induced increase was attenuated to control levels by co-treatment with L-NAME (*P *< 0.05-0.001; Figure 5C). Conversely, applying sodium nitroprusside (SNP) to MG-5 cells significantly increased Mac-1 mRNA expression in a dose-dependent manner (1.46 ± 0.09-fold in the presence of 1.0 mM SNP as compared with the non-treatment group, *P *< 0.01; Figure 6A). SNP also increased mRNA expression levels of IL-6, TLR4 and P2X4 receptor in a dose-dependent manner (10.8 ± 0.60-, 1.94 ± 0.14- and 1.67 ± 0.08-fold in the presence of 1.0 mM SNP as compared with the non-treatment group; *P *< 0.001 each; Figure 6B-D).

## Discussion

The present study provides the first evidence that n/i/eNOS triple gene knockouts attenuate peripheral nerve injury-induced tactile allodynia and spinal microglial activation. Moreover, activating the NOS/NO pathway can promote expressions of microglial IL-6, TLR4 and P2X4 receptor, which are implicated in neuropathic pain [2-6].

Chronic pain is categorized into inflammatory pain, induced by peripheral tissue inflammation, and neuropathic pain, causing damage and malfunction of the nerve system. The former can have adequate pain control by known analgesics, but the latter shows resistance to existing treatments. We demonstrated that *iNOS^-/-^*mice showed suppression of formalin-induced acute and persistent pain, but not nerve injury-induced neuropathic pain as compared with WT mice. This result in thermal hyperalgesia is consistent with a previous report showing no attenuation of the nerve injury-induced thermal hyperalgesia in iNOS-deficient mice [16]. In contrast to the behavioral phenotype of *iNOS^-/-^*mice in the formalin test, the reduction of nociceptive behaviors of *n/i/eNOS^-/-^*mice was weak in the late second phase, and in the first phase, *n/i/eNOS^-/-^*mice facilitated the nociceptive response. However, in a model of neuropathic pain, tactile allodynia induced by nerve injury was markedly inhibited only in *n/i/eNOS^-/-^*mice. These results suggest that NOS/NO signaling critically contributes to both tissue injurious pain and neuropathic pain, and that each NOS isoform has a different function in pain signaling. In previous studies, N-nitro-L-arginine, a non-selective NOS inhibitor, reduces first phase nociceptive behavior evoked by formalin, whereas the iNOS inhibitor aminoguanidine and the nNOS inhibitor 7-nitroindazole do not have a suppressing effect on the first phase pain response [26]. Furthermore, an antisense oligodeoxynucleotide targeting the nNOS-2 isoform, one of 10 splicing differentiations, has a significant exacerbation effect against the first phase [27]. While the responses of innocuous mechanical stimuli were indistinguishable between WT mice and the two genotypes of *NOS^-/-^*mice, the latency of tail flick away from noxious heat stimuli was extended in *iNOS^-/-^*mice, but not *n/i/eNOS^-/-^*mice. Acetic acid-induced visceral pain was also enhanced in *n/i/eNOS^-/-^*mice when compared with WT mice. L-NAME has been reported to have no effect on tail-flick test latencies, but L-NAME and 7-nitroindazole inhibit acetic acid-induced writhing behavior [28]. However, these previous reports illustrate the complexity of NOS in pain perception; our data lead to the hypothesis that nNOS and/or eNOS may have a role in suppressing pain signaling.

Activation of spinal microglia is known to occur after nerve injury [2,21,22]. Because minocycline, which inhibits microglial activation *in vivo *[29], reduces tactile allodynia on nerve injury [30], the microglial activated restraint leads to pain relief in neuropathic pain states. The activation of spinal microglia as well as tactile allodynia after nerve injury was suppressed in only *n/i/eNOS^-/-^*mice. The suppression of nerve injury-induced neuropathic pain responses in all NOSs-knockout mice correlated with the reduction in the numerical and morphological changes of microglia after nerve injury. These results suggest that NOS/NO signaling participates in neuropathic pain through activation of microglia.

We have previously shown that SFKs are activated in the spinal cord after nerve injury. The SFKs are the largest family of nonreceptor tyrosine kinases and nine subtypes have been identified: Src, Yes, Fyn, Fgr, Lck, Hck, Blk, Lyn and Frk [31]. We have also showed previously that Lyn is predominantly expressed in microglia. Because transcriptional activity of microglial P2X4 receptor mRNA is reduced in a neuropathic pain model using *Lyn^-/-^*mice [23], the increase of spinal p-SFKs may result in an enhancement of microglial transcriptional activity in response to nerve injury. After spinal nerve injury, activated microglia upregulate the expression of P2X4R, IL-6, IL-1β, TNF-α and TLR4 [3-5]. In fact, IL-6 and TLR4 mRNA transcriptional activities increased after nerve injury, and these increases were inhibited in *n/i/eNOS^-/-^*mice, which observed a significant degrease of p-SFKs immunoreactivity. P2X4R mRNA expression was also tendency to be reduced in *n/i/eNOS^-/-^*mice. It is possible that NOS/NO signaling may be involved in expression of these cytokine/receptor transcripts, presumably through microglial SFK activation after spinal nerve injury. It was reported that the intrathecal administration of the anti-IL-6 antibody attenuates nerve injury-induced tactile allodynia and microglial activation in rats [32]. Additionally, spinal nerve injury-induced tactile allodynia and microglial activation are inhibited in *TLR4^-/-^*mice [3]. IL-6 and TLR4 have been implicated in neuropathic pain conditions. Therefore, it is conceivable that suppressing expression of these molecules in microglia may be involved in the attenuation of nerve injury-induced tactile allodynia observed in *n/i/eNOS^-/-^*mice.

Expression of NOS and NO are known to be markedly upregulated in damaged nerves, dorsal root ganglia and glial cells in the spinal dorsal horn after nerve injury [33,34]. Our pharmacological procedure indicated the chronic intraperitoneal administration of L-NAME suppressed the development of tactile allodynia. Furthermore, acute administrations of L-NAME or 7-nitroindazole reversed established tactile allodynia on day 7 after nerve injury. It has been reported that nNOS knockout mice can attenuate neuropathic pain [15]. Because a previous study has shown that intracerebroventricular administration of 7-nitroindazole has no effect on alleviating pain in the chronic constriction injury model [13], nNOS in the brain may have a minor role in nerve injury-induced allodynia. These results suggest that NOS/NO signaling plays a key role in neuropathic pain states, and spinal or peripheral nNOS plays an important role in tactile allodynia during development and maintenance. There is no evidence that eNOS precisely contributes to neuropathic pain, although eNOS expression is upregulated in the injured sciatic nerve [35].

IFN-γ is reported to induce microglial activation and neuropathic pain through Lyn and P2X4 receptor signaling [24]. In our *in vitro *study, expression of iNOS and nNOS mRNA were increased in IFN-γ-treated MG-5 cells. In IFN-γ- or SNP-treated MG-5 cells, the significant increase in the expression of Mac-1, known as a marker of activated microglia, were observed. The IFN-γ-induced increase in Mac-1 mRNA expression was prevented by L-NAME co-treatment. From these results, it is possible that IFN-γ upregulates iNOS and nNOS expression, which in turn promotes activation of microglial cells (although it is likely that the role of iNOS in neuropathic pain may be relatively minor because a marked attenuation of allodynia was not observed in *iNOS^-/-^*mice compared with *n/i/eNOS^-/-^*mice). IL-6 and TLR4 expression, which were attenuated in *n/i/eNOS^-/-^*mice with spinal nerve injury, and P2X4 receptor mRNA were increased following SNP treatment. Upregulation of the P2X4 receptor in microglia is an important process in producing neuropathic pain [2]. *P2rx4^-/-^*mice do not show nerve injury-induced tactile hypersensitivity [36]. Thus, in addition to IL-6 and TLR4, the P2X4 receptor may also be involved in the suppression of tactile allodynia in *n/i/eNOS^-/-^*mice.

## Conclusions

In summary, we demonstrate that the NOS/NO pathway plays an important role in the development and maintenance of neuropathic pain after nerve injury. Pharmacologic and genetic studies indicate that spinal and/or peripheral NOS, especially nNOS, contributes to upregulation of IL-6, TLR4 and P2X4 receptor expression through microglial SFK activation. These findings are conducive to the understanding of the neuropathic pain mechanism.

## Methods

### Animals

All experimental procedures were performed in accordance with institutional guidelines at Kyushu University regarding the care and use of animals. Male iNOS knockout mice (*iNOS^-/-^*mice), male n/i/eNOS triple knockout mice (*n/i/eNOS^-/-^*mice) and male C57BL/6J WT mice (the genomic status of each mouse was checked) were used in the present study [20]. Male C57BL/6J mice (25-35 g) were bought from Japan SLC (Hamamatsu, Japan). Mice were housed at a constant temperature of 22 ± 1°C with a 12-hour light-dark cycle (lights on between 08:30 and 20:30), and had ad libitum access to food and water.

### Behavioral assays for acute and chronic pain

In the test of formalin-induced pain, mice were injected intraplantarly with formalin (5% (v/v), 20 μL), and the duration of licking and biting responses to the injected hindpaw were recorded at 5 min intervals for 60 min after injection (formalin pain) [36]. Noxious heat-evoked tail and hindpaw withdrawal responses were detected by the application of radiant heat (Ugo Basile, Italy) to the tail and the plantar surface of the hindpaw, respectively [36]. The intensity of the heat stimulus was adjusted to 30 or 50 V, and the latency of the tail withdrawal response (sec) was measured. The sensitivity to mechanical stimuli was assessed using von Frey filaments (0.02-2.0 g, Stoelting, Wood Dale, IL, USA), and the mechanical stimulus producing a 50% PWT was determined using the up-down method [37,38]. In the chemical visceral pain test, mice were injected intraperitoneally with acetic acid (0.8% (v/v)), and the number of abdominal writhes was counted for 5 min starting from 5 min after the injection [36].

### Spinal nerve injury-induced neuropathic pain model

The spinal nerve injury model in mice was produced as described previously [23,39]. For the experiments using mice, the left fifth lumbar spinal nerve of mice was transacted under 2% (v/v) isofluorane anesthesia.

### Immunohistochemistry

The mice were anesthetized by pentobarbital (100 mg/kg, intraperitoneally) and perfused transcardially with 4% (w/v) paraformaldehyde. The fifth lumbar segment of the lumbar spinal cord was quickly removed, postfixed in the same fixative, and placed in 30% (w/v) sucrose solution for 24 hrs at 4°C. Immunohistochemistry and analysis of transverse spinal cord sections (30 μm) were performed in accordance with methods described previously [40]. The rabbit anti-ionized calcium binding adaptor molecule 1 (anti-Iba1) antibody (1:2,000, Wako, Osaka, Japan) was used as the primary antibody, and the anti-rabbit immunoglobulin G (IgG)-conjugated Alexa Fluor 488 (1:1,000, Molecular Probes, Eugene, OR, USA) was used as the secondary antibody. The intensity of p-SFKs immunoreactivity was analyzed using a software Photoshop CS3 (Adobe, San Jose, CA).

### Drug administration

Phosphate buffered saline (PBS; as a vehicle control) or L-N^G^-nitro-arginine methyl ester (L-NAME) (Sigma, St. Louis, MO, USA; 30 or 100 mg/kg) was intraperitoneally administered to rats once a day for 7 days with a 26 G needle. Measurement of PWT was performed on 0, 1, 3, 5 and 7 days after administration. For intrathecal drug administration, under 2% (v/v) isofluorane anesthesia, mice were injected with a 32 G needle. After peripheral nerve injury, rats were administered intrathecally with PBS (5 μL, as a vehicle control) or L-NAME (100 mg/kg) at day 7 after nerve injury. Measurement of PWT was performed at 0.5, 1, 1.5, 2, 3, 6 and 12 hrs after administration. Similarly, 7-nitroindazole (Calbiochem, Darmstadt, Germany; 3 or 10 μg in 5 μL) was intrathecally administered and measurement of PWT was performed at 0.5, 1, 1.5, 2 and 3 hrs after administration. Intrathecal drug administration was performed just after pre-measurement, and PWT was measured at each time point.

### MG-5 Culture

The MG-5 mouse microglial cell line was cultured as described previously by Ohsawa et al. [25]. Conditioned medium from the supernatant of A1 cells cultured overnight in Dulbecco's modified Eagle's medium (Invitrogen, Carlsbad, CA, USA) containing 10% (v/v) fetal bovine serum, penicillin and streptomycin (20 U/mL) was used as the culture medium for MG-5 cells. MG-5 cells were exposed to IFN-γ and SNP for 1 hr under serum-free conditions.

### Real-time reverse transcription-PCR

The fifth lumbar spinal cord was quickly removed. Total RNA preparation and real-time RT-PCR amplification/detection were performed in accordance with methods described previously [40]. All expression values were normalized by the expression values of 18S ribosomal RNA. The TaqMan probe and the forward and reverse primers used in this study were designed according to Table 1. Ribosomal RNA was measured using TaqMan Ribosomal RNA Control Reagents (P/N 4308329, Applied Biosystems, Carlsbad, CA, USA). The TaqMan probe and primers for Mac-1 were obtained from Applied Biosystems.

**Table 1:** Sequences of TaqMan probe, forward primer and reverse primer of nNOS, iNOS, IL-6, TLR4 and P2X4R for real time quantitative RT-PCR

	probe and primers	Sequence
nNOS	probe	5'-FAM-TTGGCCCAGGCACCGGCA-TAMRA-3'
	forward primer	5'-TTCCACCTGCCTCGAAACC-3'
	reverse primer	5'-GCACGTCCTGTACATATTTCTTTGG-3'
iNOS	probe	5'-FAM-CCCCCAGCGGAGTGACGGC-TAMRA-3'
	forward primer	5'-ACATCAGGTCGGCCATCACT-3'
	reverse primer	5'-CGTACCGGATGAGCTGTGAAT-3'
IL-6	probe	5'-FAM-TCACAGAGGATACCACTCCCAACAGACCTG-TAMRA-3'
	forward primer	5'-GGGACTGATGCTGGTGACAA-3'
	reverse primer	5'-TGCCATTGCACAACTCTTTTCT-3'
TLR4	probe	5'-FAM-CACGTCCATCGGTTGATCTTGGGAGAA-TAMRA-3'
	forward primer	5'-AAACTTGCCTTCAAAACCTGGC-3'
	reverse primer	5'-ACCTGAACTCATCAATGGTCACATC-3'
P2X4R	probe	5'-FAM-CAATGAGCAACGCACACTCACCAAGG-TAMRA-3'
	forward primer	5'-ACAACGTGTCTCCTGGCTACAAT-3'
	reverse primer	5'-GTCAAACTTGCCAGCCTTTCC-3'

### Statistical analysis

Data are expressed as the means ± SEM. Statistical analyses of the results were conducted with one-way ANOVA, one-way repeated ANOVA, two-way ANOVA or two-way repeated ANOVA with a *post hoc *test (Tukey's multiple comparison test). Statistical significance was set at a *P *value < 0.05.

## Competing interests

The authors declare that they have no competing interests.

## Authors' contributions

KK performed the experiments, analyzed the data and wrote the manuscript; MTsuda designed, performed and supervised the experiments, and wrote the manuscript; MTsutsui, YT, HS and NY provided *iNOS^-/-^*and *n/i/eNOS^-/-^*mice, and edited the manuscript; HT-S supervised the experiments and analyzed the data; KI coordinated the project, helped to interpret the data, and edited the manuscript. All authors have read and approved the final manuscript.
